# Information Content-Based Gene Ontology Functional Similarity Measures: Which One to Use for a Given Biological Data Type?

**DOI:** 10.1371/journal.pone.0113859

**Published:** 2014-12-04

**Authors:** Gaston K. Mazandu, Nicola J. Mulder

**Affiliations:** 1 Computational Biology Group, Department of clinical Laboratory Sciences, IDM, University of Cape Town Faculty of Health Sciences, Cape Town, South Africa; 2 African Institute for Mathematical Sciences (AIMS), Cape Town, South Africa, and Cape Coast, Ghana; University of North Carolina at Charlotte, United States of America

## Abstract

The current increase in Gene Ontology (GO) annotations of proteins in the existing genome databases and their use in different analyses have fostered the improvement of several biomedical and biological applications. To integrate this functional data into different analyses, several protein functional similarity measures based on GO term information content (IC) have been proposed and evaluated, especially in the context of annotation-based measures. In the case of topology-based measures, each approach was set with a specific functional similarity measure depending on its conception and applications for which it was designed. However, it is not clear whether a specific functional similarity measure associated with a given approach is the most appropriate, given a biological data set or an application, i.e., achieving the best performance compared to other functional similarity measures for the biological application under consideration. We show that, in general, a specific functional similarity measure often used with a given term IC or term semantic similarity approach is not always the best for different biological data and applications. We have conducted a performance evaluation of a number of different functional similarity measures using different types of biological data in order to infer the best functional similarity measure for each different term IC and semantic similarity approach. The comparisons of different protein functional similarity measures should help researchers choose the most appropriate measure for the biological application under consideration.

## Introduction

The advancement of high-throughput biology technologies has resulted in a large increase in functional data, eliciting the need for relevant tools that help analyze and extract information from these data. The Gene Ontology (GO) [1] is an established standard for the functional annotation of proteins that successfully provides structured and controlled, organism-independent vocabularies to describe gene functions and a well adapted platform to computationally process data at the functional level [2]. Currently, several proteins are already annotated with GO terms in the existing biological databases [3]–[6], thus enabling protein comparisons on the basis of their GO annotations. Even though the high proportion (more than 98%) of these annotations are inferred electronically (mostly based on transitive mappings from InterPro2GO, SPKW2GO, EC2GO, SPSL2GO, HAMAP2GO and UniPathway2GO), with IEA (Inferred from Electronic Annotation) as the GO evidence code (http://www.geneontology.org/GO.evidence.shtml), these annotations are becoming more and more accurate with an increased level of confidence as the different mappings are manually curated [7].

Several functional similarity measures that quantify similarity between proteins based on their GO annotations have been introduced and successfully applied in many biomedical and biological applications [2], [8]. These measures allow the integration of the biological knowledge contained in the GO structure [9], and have contributed to the improvement of biological analyses [2]. These measures are derived either directly from the GO term information content (IC), a numerical value scoring the description and specificity of a GO term using its position in the GO directed acyclic graph (DAG), or from GO term semantic similarity scores conveying information shared by two GO terms in the GO DAG [8]. It is worth mentioning that several term semantic similarity models have been introduced and a detailed review can be found in [10], [11]. In this study, we are only focusing on term semantic similarity models that are based on term information content, known as node-based models [8], [11]. In order to quantify the information content (IC) value of a given term, several approaches have also been proposed, each depending on how the concept ‘specificity’ is conceived in the context of the GO DAG structure. These approaches are partitioned into two main families, namely annotation- and topology families, and have been largely used to compare GO terms in the GO DAG and proteins at the functional level using their GO annotations.

The annotation family uses GO term statistics in the corpus under consideration. Despite the issue of protein annotation dependence (scores are based on annotation, which may be unbalanced, biased and incomplete), which leads to shallow annotation problem [10] that affects semantic similarity scores produced [12], this family has been used in several applications. Several approaches for comparing GO terms have been tested in the context of the GO DAG, the most popular node-based semantic similarity approaches include the Resnik [13], Lin [14] and Jiang & Conrath [15] approaches, which were initially suggested in the context of the WordNet and adapted to the GO DAG [16]. Recently, the Nunivers approach [8] has been introduced and different enhancements, such as Disjunct Common Ancestor (DCA) [17], relevance similarity [18], information coefficient similarity [19] and eXtended GraSM (XGraSM) [8] model were proposed to improve the existing approaches for GO term comparison. Note that a random walks enhancement [20] was proposed to improve any of the existing similarity measures by modeling inherent uncertainty from the incomplete knowledge of gene annotations and ontology structure. Functional similarity measures induced by GO term semantic similarity approaches include average (Avg) [16], maximum (Max) [21], average of the best matches (ABM) [2], and best match average (BMA) [9], and those using the GO term information content directly, namely SimGIC [22], SimUI [23], SimUIC and SimDIC [2], [9].

The topology-based family, which only uses the structure of the GO DAG in the computation of the IC values, has been proposed to correct for the effect of annotation dependence and provide an effective way of measuring functional similarity between proteins based on their GO annotations. The earliest type of topology-based family, namely edge- or path-based semantic similarity measures, suffers from a serious drawback of producing uniform scores for terms at the same level of the hierarchy under consideration as these scores are obtained using path lengths between terms [8]. These measures ignore the position characteristics of terms in the hierarchy and a solution based on differently weighting edges was suggested, but failed to completely resolve the problem [9], [11]. In this study, we are only considering the node-based approaches as pointed out previously, which use the concept of IC score to compare the properties of the terms themselves and relations to their ancestors or descendants, and taking into account term position characteristics [9]. These measures are referred to as IC-based approaches and overcome the main issue of edge- or path-based approaches, producing a fixed and well defined IC score for a given GO term, independent of the corpus or source under consideration. Each topology-based approach provides its specific semantic similarity measure for comparing GO terms, and functional similarity measure for scoring protein closeness. However, none of the existing studies has attempted to evaluate the effectiveness of functional similarity measures proposed in the context of the annotation-based approaches when applied to the topology-based approaches. Such a study is important to determine the most appropriate functional similarity measure for each approach given the biological application.

Here, we investigate the behaviour of several different IC-based functional similarity measures suggested in the context of annotation-based and topology-based approaches, using different biological data, including protein-protein interaction networks, protein domain and other functional data. Each measure performs differently for different applications [2] and interprets the DAG structure of the GO differently [8], [9]. Thus, one needs to understand these differences in order to choose an effective measure for analysis of a dataset, which can be cumbersome and tedious for someone who just needs a quick GO semantic similarity measure for their biological question. This suggests that the quantitative comparative study of all existing GO semantic similarity measures and approaches is necessary to enable one to quickly identify the most effective measure, among the several semantic similarity tools available, for their application. This study provides a mapping between a term IC or term semantic similarity approach and its corresponding most ‘appropriate’ functional similarity measure, given a particular biological application.

## Materials and Methods

To evaluate the existing IC-based functional similarity measures which have been used in the context of biomedical and bioinformatics applications, we use different functional data, including protein sequence, Pfam domain and enzyme commission (EC) similarity data, human gene expression (microarray) and protein-protein interaction (PPI) datasets. All these data represent some form of ‘grouping’ of proteins that should be functionally related and thus provide useful tests for GO similarity measures. The complete set of GO data and protein-GO term associations were extracted from the GO and GOA databases, respectively, released on the 15th April, 2014. We have considered three topology-based approaches, namely the GO-universal metric proposed by Mazandu and Mulder [9], and the methods of Wang et al. [24] and Zhang et al. [25]. In general, the information content (IC) or semantic value of a given term t is computed as follows: 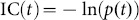
(1)where 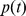 is the relative frequency of occurrence of the term 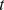 in the protein annotation dataset under consideration [16], which is the D-value [25] and topological position characteristic of 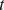 in the context of annotation family, the Zhang and GO-universal approaches, respectively. Note that the Zhang et al. model for computing the IC score follows the Seco et al. approach [26] in its conception and it is adapted to the context of the GO-DAG. For the Wang et al. method, the IC score of a given term 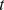 is the sum of S-value of the term 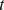 and those of all its ancestors [24]. The term semantic similarity score 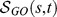 between GO terms 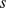 and 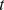 can be retrieved from the following formula [8]: 
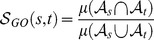
(2)where 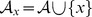 and 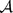 denotes the set of ancestors of the term 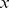, 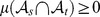 and 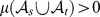 are measures of the commonality between and of the description of 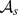 and 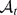, respectively. The formula 2 is a unified formula of all term semantic similarity models based on IC or SV values of terms. Note that other term semantic similarity models that do not use only or directly IC values were proposed. These include the Hybrid Relative Specificity Similarity (HRSS) method [27], which adapts both node- and edge-based concepts, and the Shortest Semantic Differentiation Distance (SSDD), which assesses the distance between terms in the GO DAG in order to measure their semantic similarity score [28], and these methods are beyond the scope of this study.

### Measuring protein similarity at the functional level

Several measures have been proposed for estimating functional similarity scores in the context of annotation-based IC approaches to facilitate protein comparisons at the functional level. These functional similarity scores are obtained using statistical measures of closeness, such as average (Avg), maximum (Max), best-match average (BMA) and averaging all the best matches (ABM). The average and maximum measures are computed as follows: 
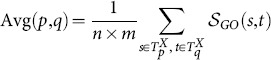
(3)and 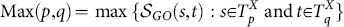
(4)where 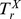 is a set of GO terms in 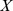 representing the molecular function (MF), biological process (BP) or cellular component (CC) ontology annotating a given protein 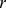 and 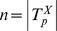 and 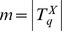 are the number of GO terms in these sets, and 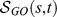 is the semantic similarity score.

The ABM [2] for two annotated proteins is the mean of best matches of GO terms of each protein against the other, given by the following formula: 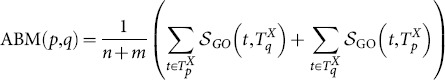
(5)with 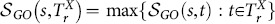. The Best Match Average (BMA) [2], [9] for two annotated proteins 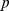 and 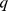 is the mean of the following two values: average of best matches of GO terms annotated to protein 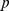 against those annotated to protein 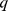, and average of best matches of GO terms annotated to protein 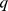 against those annotated to protein 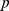, given by the following formula: 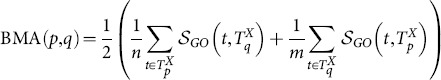
(6)


Note that the four functional similarity measures above require GO term semantic similarity scores, and are referred to as IC-based non-direct term or term semantic similarity- or pair-wise term-based measures [2]. For the topology-based family, each approach has been suggested with its functional similarity measure. The GO-universal metric [9] uses BMA, and ABM was used in the Wang et al. approach [24]. The Zhang et al. measure [25] is a context dependent approach and authors initially suggested using the approach proposed by Lord et al. [16], which is the Avg scheme for measuring functional similarity scores between proteins.

In the context of the annotation-based family, it has been observed that measuring the semantic similarity of two GO terms based only on the most informative common ancestor terms cannot discern the semantic contributions of the ancestor terms to these two specific terms and thus may negatively impact functional similarity scores. The GraSM and XGraSM approaches have been proposed and shown to perform better than those using only the most informative common ancestors (MICA) strategy [8]. This argument has been confirmed through the performance evaluation of the SimGIC measure suggested by Pesquita et al. [22], which uses a Jaccard index weighted by IC of terms, thus incorporating the features of all ancestors of the terms. The SimGIC measure computes the functional similarity score between two proteins 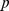 and 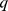 as follows: 
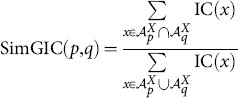
(7)where 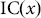 is the information content value of the term 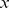 [8] and 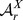 a set of GO terms together with their ancestors in 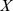 representing the ontology (MF, BP or CC) annotating a given protein 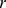.

Using the observation above, we proposed two other possible functional similarity schemes [2], [9], using Dice (Czekanowski or Lin like measure) and universal indexes, referred to as SimDIC and SimUIC, respectively, and given by the following formulae: 
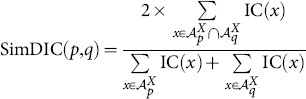
(8)

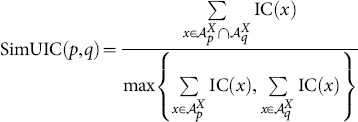
(9)


Note that this study provides the first evaluation of these SimDIC and SimUIC measures and their comparison to other functional similarity measures. Unlike the Avg, Max, ABM and BMA measures, in which semantic similarity between GO terms is required in the computation of functional similarity scores, the SimGIC, SimDIC and SimUIC measures use the IC of terms directly and they are referred to as IC-based direct term measures. Note that there exist other functional similarity models, such as shortest-path graph kernel (spgk) [29], using the intrinsic topology of the GO DAG for directly estimating protein functional similarity scores without computing the IC scores of GO terms or semantic similarity scores between terms. Here, we are only focusing on protein functional similarity models that use the IC of terms.

### Assessing different functional similarity measures

We systematically assess different functional similarity measures on different types of functional data, including sequence similarity, Pfam domain and Enzyme Commission (EC) similarity data on a selected set of proteins, and human protein-protein interaction (PPI) and co-expression networks. These datasets represent different types of biological data used to evaluate GO semantic similarity measures [10]. Depending on these biological data, different performance measures are used to elucidate the ‘best’ semantic similarity measure or approach.

### Correlation with EC, Pfam and sequence similarity

Generally, the comparison of different semantic similarity measures is performed using Pearson's correlation measures with sequence, Pfam domain and Enzyme Commission (EC) similarity data. This correlation provides an indication of how effective the functional similarity measure is in capturing sequence, Pfam, and EC similarity. This means that a measure with a higher correlation is better, since it captures these similarities well and it is likely to be an unbiased measure. To compare different measures, we ran the Collaborative Evaluation of Semantic Similarity Measures (CESSM) online tool [30] at http://xldb.di.fc.ul.pt/tools/cessm/ for BP and MF using a dataset of selected proteins with known relationships downloaded from the CESSM website.

### Performance evaluation using a PPI network

Different measures were assessed in terms of their ability to capture functional coherence in a human PPI network based on how interacting proteins are functionally related to each other. Human PPI datasets were downloaded from several different PPI databases, including the IntAct, DIP, BIND, MIPS, MINT and BioGRID databases, and integrated into a single network in which only interactions predicted by at least two different approaches and found in the STRING dataset are considered, to reduce the impact of false positives. This produced a human PPI network with 6031 interactions from which a total of 5366 and 5580 interactions with both interacting partners were among 29844 and 31683 proteins annotated with respect to the GO BP and CC ontologies, respectively. These interaction datasets are available in the supplementary data (see Tables S1, S2 and S3 in File S1) and can also be downloaded from the CBIO website at http://web.cbio.uct.ac.za/ITGOM/funcsimdata.

The set of these 5366 and 5580 interactions are considered as a positive set, while the negative set consists of the same number of interactions randomly selected among annotated human proteins pairs. This is consistent as the chance of randomly selecting a detected PPI is very small (less than 0.0012%). We only considered proteins annotated with BP and CC terms in the network produced since two proteins that interact physically are more likely to be involved in similar biological processes or localized in the same cellular component, but there is no guarantee that they share molecular functions [9]. The classification power of different functional similarity measures was tested using Receiver Operator Characteristic (ROC) curve analysis, which assesses the Area Under the Curve (AUC), plotting the true positive rate or sensitivity vs the false positive rate or 1-specificity. This AUC value is used as a measure of discriminative power and a realistic classifier must have an AUC larger than 0.5.

### Clustering power on a gene expression dataset

We use the human co-expression network retrieved from the Bossi et al. [31] and the STRING human network. We retrieved 7228 co-expressed protein pairs of which a total of 6995 pairs have both proteins found among 29844 human proteins annotated with BP terms (see Tables S4 and S5 in File S1, or go to http://web.cbio.uct.ac.za/ITGOM/funcsimdata). We are only considering the BP ontology as co-expressed genes are more likely to share common processes and may at least belong to the same pathway or contribute to a similar biological process [32]. We partitioned these co-expressed proteins into different clusters using the Blondel et al. method [33] and the corresponding partition is considered to be a ground truth, i.e., the true partition of the actual co-expressed network. Thereafter, the interactions from the co-expressed network are weighted using functional similarity scores and proteins clustered using the same clustering method. We assessed the clustering power of a given functional similarity measure by comparing this clustering result to the ground truth using Normalized Mutual Information and Rank Index of pairwise cluster memberships [34].

Let 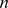 be the number of proteins in the network with the ground truth (g) having p partitions, each with 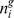 proteins, 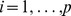, and clustering result (c) with q partitions, each with 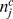 proteins, 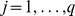. The entropy 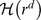 of a given clustering (d) having r partitions, each with 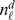 proteins, 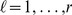, is given by: 
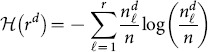
(10)and the mutual information 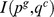 between the two partitions is computed as follows: 
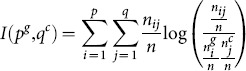
(11)where 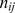 is the number of common proteins between the 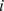
th cluster in the ground truth and the 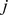
th cluster in the clustering result. This implies that the normalized mutual information 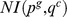 is given by: 
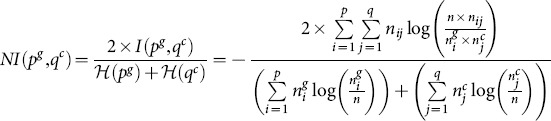
(12)


Finally, the Rank Index 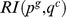 of pairwise cluster memberships is computed as follows: 
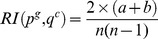
(13)where 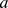 is the number of pairs of proteins belonging to the same cluster in the ground truth and clustering result, and 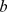 the number of protein pairs belonging to different clusters in the ground truth and clustering result. The functional similarity measure providing higher normalized mutual information and accuracy scores is considered to be the ‘best’ one.

## Results and Discussion

Previous work on semantic similarity measures has suggested that the appropriate use of functional similarity measures depends on the biological applications and different measures perform differently for different applications [2]. Each semantic similarity approach or functional measure was defined for a specific purpose with a specific application in mind, especially in the context of topology-based approaches, where each approach was set with its specific functional similarity measure, depending on its conception and the applications for which it was designed. These applications include, protein-protein interaction assessments, protein function prediction, protein clustering, etc. and results were often tested against the expectations of the performance scores. Here, we assess the performance of different measures on different biological applications or data, including EC, Pfam domain and sequence similarity on a selected set of protein pairs, and human PPI and co-expression network or expression data, in order to elucidate the most ‘appropriate’ measures for different approaches and biological applications. The summary of different approaches that are combined to construct 57 different IC-based functional similarity measures used is provided in Table 1. Note that the Jiang and Conrath approach is not used explicitly since it has been shown to be a particular case of the Lin approach [8].

**Table 1:** Summary of different IC-based functional similarity and term semantic similarity measures.

Measure	Model	Approach	Reference
Functional similarity	IC-based direct term	SimGIC	[22]
		SimDIC	[2]
		SimUIC	[2]
		SimUI	[23]
	Pair-wise term or IC-based non direct term	Avg	[16]
		Max	[21]
		BMA	[22]
		ABM	[24]
Term Semantic Similarity	Annotation-based	Resnik	[13]
		XGraSM-Resnik	[8]
		Nunivers	[8]
		XGraSM-Nunivers	[8]
		Lin	[14]
		XGraSM-Lin	[8]
		Li et al.	[19]
		Relevance	[18]
	Topology-based	GO-Univeral	[9]
		Wang et al.	[24]
		Zhang et al.	[25]

These measures were used to built 57 different functional similarity measures that are assessed using different types of biological data, including Enzyme Commission (EC), Pfam domain, Sequence Similarity (Seq. Sim.), Protein-Protein Interaction (PPI) and Co-expression Network (CN) or Gene Expression (microarray) data.

### Using EC, Pfam and Sequence Similarity data

**Figure 1:**
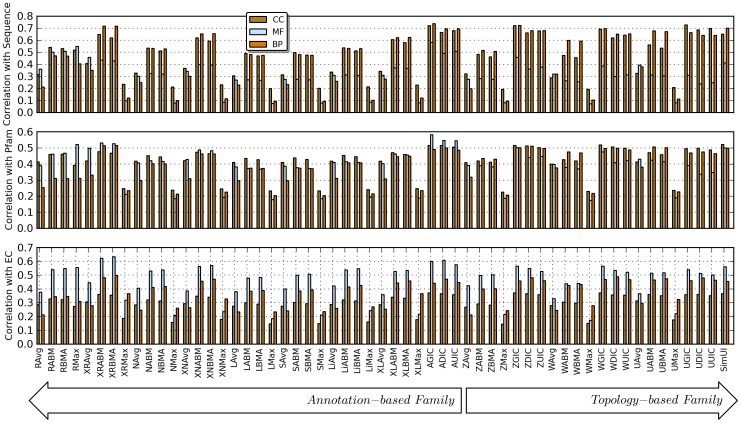
Performance evaluation in terms of Pearson's correlation values. These different Pearson's correlation values with Enzyme Commission (EC), Pfam and Sequence similarity are obtained from the CESSM online tool. For x-axis labels, the prefixes R, N, L, Li, S, X, A, Z, W, and U represent the approaches and stand for Resnik, Nunivers, Lin, Li, Relevance, XGraSM, Annotation-based, Zhang, Wang and GO-universal, respectively. The suffixes GIC, UIC and DIC represent SimGIC, SimUIC and SimDIC measures, respectively. In cases where the prefix X is used, it is immediately followed by the approach prefix. Refer to Table 2 and 3 for the description of these different measures.

We used a dataset of proteins with known relationships downloaded from the CESSM online tool. The GO annotations of different proteins in the dataset were retrieved from the GOA-UniProtKB dataset. The CESSM tool has made the comparison of different functional similarity measures using Pearson's correlation measures with sequence, Pfam domain and EC similarity possible. We ran the CESSM online tool and results are shown in Figure 1 for the BP, MF and CC ontologies. Except for the Resnik approach, these results show that in general there is a good correlation between EC, Pfam domain, sequence similarity and functional similarity measures for BP, MF and CC, especially when using measures other than Max and Avg. For EC in particular, the MF ontology tends to display higher levels of correlation. This is unsurprising as EC numbers are very specific for a particular function, so there should be good correlation in MF terms.

Recently, it was shown that the normalization model and correction factors have an impact on the performance of functional similarity measures [8]. It is likely that the effect of the normalization factor is a serious drawback of the Resnik approach as this has an impact on its performance and makes it inconsistent with the hierarchy under consideration. This is confirmed by looking at the performance of the Nunivers [8] and Lin [14] approaches (see Table 2), which follow the general pattern, whereas the Resnik approach suggests the Max measure for the MF ontology. In general, BMA and ABM measures provide the best performance and they perform equally in most cases. On the other hand, the use of an efficient correction factor may improve a given approach or measure. If the information coefficient and relevance introduced by Li et al. [19] and Schlicker et al. [18], respectively, which use the IC value of the most informative common ancestor between terms, does not significantly improve the performance of the Lin approach, then one can consider all common informative ancestors in the correction factor to enhance the performance of the approach [8].

**Table 2:** Pearson's correlation values of different measures.

Approach	Measure	Molecular Function (MF)			Cellular Component (CC)			Biological Process (BP)		
		EC	PFAM	Seq Sim	EC	PFAM	Seq Sim	EC	PFAM	Seq Sim
R	Avg (RAvg)	0.37532	0.38905	0.36071	0.28501	0.41217	0.31369	0.21121	0.25342	0.21123
	ABM (RABM)	0.53917	0.46098	0.50052	0.32731	0.45986	0.54093	0.34236	0.31019	0.46918
	BMA (RBMA)	0.54787	0.46651	0.50675	0.32117	0.46045	0.52959	0.34478	0.30893	0.46692
	Max (RMax)	0.55531	0.52123	**0.54861**	0.27177	0.39069	0.51783	0.30957	0.30944	0.40344
	XGraSM-Avg (XRAvg)	0.44532	0.49719	0.45670	0.30590	0.41945	0.40519	0.27856	0.32978	0.35064
	XGraSM-ABM (XRABM)	0.62228	**0.53063**	0.43396	**0.35963**	**0.47602**	**0.64791**	0.48034	0.51387	0.71515
	XGraSM-BMA (XRBMA)	**0.63390**	0.52562	0.42832	0.35322	0.46763	0.61850	**0.49735**	**0.51565**	**0.71682**
	XGraSM-Max (XRMax)	0.31849	0.21078	0.09714	0.18628	0.24576	0.23365	0.36543	0.23418	0.12051
N	Avg (NAvg)	0.40437	0.40605	0.30088	0.28388	0.41696	0.32773	0.24504	0.29716	0.24824
	ABM (NABM)	0.52989	0.42083	0.32264	0.31967	0.45185	0.53365	0.40960	0.40157	0.53209
	BMA (NBMA)	0.53717	0.41589	0.31800	0.31157	0.44377	0.51035	0.41764	0.39956	0.52862
	Max (NMax)	0.20693	0.18493	0.07917	0.15753	0.23744	0.21049	0.26021	0.21171	0.10015
	XGraSM-Avg (XNAvg)	0.38562	0.42789	0.34098	0.29236	0.41989	0.36626	0.26348	0.30804	0.30011
	XGraSM-ABM (XNABM)	0.56160	**0.48742**	**0.39713**	**0.34711**	**0.47237**	**0.61938**	0.45603	0.46176	0.65137
	XGraSM-BMA (XNBMA)	**0.57036**	0.48224	0.39241	0.33988	0.46313	0.59236	**0.47026**	**0.46259**	**0.65310**
	XGraSM-Max (XNMax)	0.23379	0.19230	0.08402	0.17896	0.24475	0.22948	0.32608	0.22527	0.11304
L	Avg (LAvg)	0.37960	0.38149	0.26975	0.27358	0.40980	0.30420	0.23344	0.29618	0.22678
	ABM (LABM)	0.47794	0.37214	0.27193	0.29969	0.43507	0.49146	0.38369	0.37405	0.47976
	BMA (LBMA)	0.48346	0.36783	0.26797	0.28974	0.42621	0.46926	0.38909	0.37171	0.47449
	Max (LMax)	0.18341	0.17780	0.07476	0.14639	0.23298	0.19865	0.23248	0.20287	0.09363
	XGraSM-Avg (XLAvg)	0.35730	0.40170	0.30816	0.28454	0.41735	0.34196	0.25193	0.30720	0.27799
	XGraSM-ABM (XLABM)	0.52692	**0.46197**	**0.37084**	**0.34015**	**0.46936**	**0.60438**	0.44261	0.44548	0.62035
	XGraSM-BMA (XLBMA)	**0.53391**	0.45679	0.36643	0.33214	0.45976	0.57860	**0.45741**	**0.44757**	**0.62307**
	XGraSM-Max (XLMax)	0.21668	0.18726	0.08100	0.17668	0.24590	0.22795	0.36543	0.23418	0.12051
S	Avg (SAvg)	0.39895	**0.38633**	**0.27616**	0.27509	0.40934	0.31267	0.24007	0.29585	0.23224
	ABM (SABM)	0.49846	0.37641	0.27502	**0.30219**	**0.43663**	**0.49769**	0.38575	**0.37302**	**0.47975**
	BMA (SBMA)	**0.50556**	0.37236	0.27109	0.29257	0.42855	0.47516	**0.39178**	0.37108	0.47462
	Max (SMax)	0.20848	0.18507	0.07914	0.14737	0.23302	0.20005	0.23424	0.20336	0.09398
Li	Avg (LiAvg)	0.42024	0.40930	0.30788	0.28658	0.41761	0.33494	0.25799	0.31039	0.25784
	ABM (LiABM)	0.53691	**0.41434**	**0.31170**	**0.32059**	**0.45221**	**0.53550**	0.41396	0.40670	**0.53182**
	BMA (LiBMA)	**0.54534**	0.41010	0.30739	0.31239	0.44524	0.51221	**0.42395**	**0.40698**	0.52966
	Max (LiMax)	0.24125	0.19425	0.08499	0.16030	0.24041	0.21243	0.26839	0.21407	0.10168
A	SimGIC (AGIC)	0.59941	**0.58159**	**0.58246**	**0.36956**	**0.51559**	**0.71940**	0.44164	0.49011	**0.73662**
	SimDIC (ADIC)	**0.60705**	0.54614	0.49134	0.36469	0.51438	0.66385	**0.46985**	**0.49947**	0.69403
	SimUIC (AUIC)	0.57433	0.54488	0.50643	0.35844	0.50424	0.67929	0.44573	0.48520	0.69341
Z	Avg (ZAvg)	0.42242	0.39074	0.27595	0.26767	0.40746	0.32121	0.21181	0.31769	0.19658
	ABM (ZABM)	0.49670	0.38912	0.28048	0.29104	0.41915	0.48201	0.39965	0.43449	0.51446
	BMA (ZBMA)	0.50184	0.38219	0.27446	0.28135	0.41131	0.46165	0.40097	0.42915	0.50697
	Max (ZMax)	0.21496	0.18623	0.08015	0.14434	0.22535	0.19262	0.24156	0.20658	0.09524
	SimGIC (ZGIC)	**0.56432**	**0.50128**	**0.45796**	**0.37052**	**0.51454**	**0.71947**	0.45672	0.50121	**0.72305**
	SimDIC (ZDIC)	0.54733	0.44010	0.36048	0.36433	0.51140	0.66128	0.48173	**0.50994**	0.67914
	SImUIC (ZUIC)	0.52587	0.44723	0.37719	0.35847	0.50159	0.67704	**0.45863**	0.49626	0.67906
W	Avg (WAvg)	0.32939	0.39711	0.31829	0.27822	0.39797	0.28790	0.24429	0.37518	0.31967
	ABM (WABM)	0.43759	0.37805	0.26197	0.30419	0.42580	0.47450	0.42471	0.47434	0.59775
	BMA (WBMA)	0.43853	0.36980	0.25558	0.29551	0.41827	0.45501	0.43182	0.46893	0.59284
	Max (WMax)	0.17071	0.17392	0.07249	0.14920	0.22981	0.19250	0.27792	0.21691	0.10267
	SimGIC (WGIC)	**0.56384**	**0.47498**	**0.38497**	**0.36989**	**0.51797**	**0.69199**	0.46808	0.49629	**0.69617**
	SimDIC (WDIC)	0.53335	0.40794	0.29754	0.35580	0.50564	0.61990	**0.48802**	**0.49858**	0.65003
	SimUIC (WUIC)	0.52018	0.42227	0.31293	0.35396	0.49725	0.64186	0.46571	0.48716	0.65164
U	Avg (UAvg)	0.36584	**0.43023**	**0.39394**	0.31186	0.41240	0.32592	0.29650	0.38034	0.37786
	ABM (UABM)	0.51354	0.42361	0.31259	**0.36012**	0.47023	0.56028	0.46424	**0.50576**	**0.67637**
	BMA (UBMA)	0.51740	0.41406	0.30507	0.35088	0.45819	0.53448	0.47364	0.50134	0.67084
	Max (UMax)	0.21967	0.18836	0.08140	0.17511	0.23499	0.20663	0.32326	0.22605	0.11111
	SimGIC (UGIC)	**0.53864**	0.39141	0.30800	0.35707	0.49532	**0.72673**	0.45891	0.46904	0.66193
	SimDIC (UDIC)	0.51113	0.33578	0.23846	0.35868	**0.49786**	0.68552	**0.47950**	0.47434	0.63920
	SimUIC (UUIC)	0.50018	0.34776	0.24722	0.35052	0.48685	0.69761	0.46210	0.46436	0.63865
SimUI	SimUI	0.56126	0.49980	0.41280	0.36520	0.52065	0.64969	0.45463	0.49754	0.69992

Comparing performance of 57 different functional similarity measures using Pearson's correlation with Enzyme Commission (EC), Pfam and Sequence similarity. Results are obtained from the CESSM online tool and the best scores are in bold. R, N, L, Li, S, X, A, Z, W, and U represent the approaches and stand for Resnik, Nunivers, Lin, Li, Relevance, XGraSM, Annotation-based, Zhang, Wang and GO-universal, respectively. The double middle bold line separates annotation-based approaches above from the topology-based approaches below.

As displayed in Figure 1 and Table 2, applying the XGraSM correction factor to the Resnik, Lin and Nunivers approaches significantly improved their performance. Thus, including common informative ancestors in the conception of a semantic similarity measure improves its performance, especially for approaches that include only the feature of child terms in the computation of IC. This is the case for the annotation-based, Zhang et al. and Wang et al. approaches, where the SimGIC measure shows an overall best performance. Note that this is not the case for the GO-universal metric, in which, the BMA measure performs better than other measures, and it also provides better performance for the Wang et approach when applied to EC data, even though the Wang et al. approach initially used the ABM measure. It follows that in the context of the annotation-based family, if one chooses to use the IC-based non-direct measures, it is advantageous to use the XGraSM enhancement model, in which case, Resnik-BMA shows overall best performance. The SimUI approach [23] refers to the union-intersection protein similarity measure and it is a particular case of SimGIC assigning equal IC value to all terms in the GO-DAG [9]. Even though this assumption is not realistic in the context of the GO DAG, the SimUI measure can still be used as an alternative measure in practice as it shows relatively good performance when applied to these different data.

### Using protein-protein interaction and expression data

**Figure 2:**
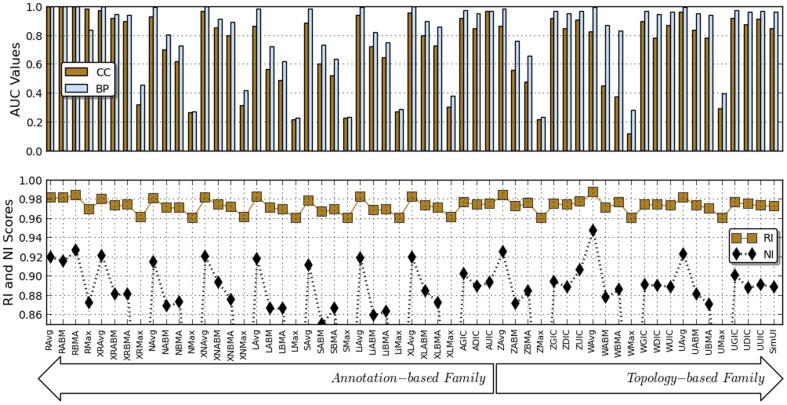
Performance evaluation in terms of clustering power (RI and NI) and Area Under the Curve (AUC) values. Different x-axis labels are the same as in Fig. 1, where different prefixes and suffixes stand for different term semantic similarity approaches and functional similarity measures.

We used human PPI and co-expressed networks to assess the performance of different functional similarity measures. In the case of the PPI network, we are using the AUC values computed using the ROCR package under the R programming language as a measure of classification power. The larger the upper AUC value, the more efficient the functional similarity measure is. For the co-expression network, we computed the NI and RI values as measures of clustering power, the higher these values, the more powerful the functional similarity measure is. Different values found for different measures are shown in Figure 2 and Table 3. These results indicate that independently of the approaches, the Avg measure, which is the earliest proposal suggested by Lord et al. [16] in the context of the IC-based functional similarity, performs better than any other functional similarity measure.

**Table 3:** Area under the curve (AUC), Rand Index (RI) and Normalized Mutual Information (NI) values of different measures.

Approach	Measure	Protein-Protein Interaction		Gene Expression	
		AUC (CC)	AUC (BP)	RI (BP)	NI (BP)
R	Avg (RAvg)	**0.9999989**	**0.9999944**	0.9814900	0.9202300
	ABM (RABM)	0.9999815	0.9997248	0.9819800	0.9159100
	BMA (RBMA)	0.9999656	0.9995277	**0.9842500**	**0.9274300**
	Max (RMax)	0.9823696	0.8355199	0.9699500	0.8729600
	XGraSM-Avg (XRAvg)	0.9715316	0.9965294	0.9804600	0.9218900
	XGraSM-ABM (XRABM)	0.9191044	0.9466970	0.9732500	0.8811700
	XGraSM-BMA (XRBMA)	0.8933883	0.9367340	0.9740800	0.8815500
	XGraSM-Max (XRMax)	0.3196787	0.4527575	0.9612000	0.7056700
N	Avg (NAvg)	0.9281535	0.9912221	0.9811400	0.9151300
	ABM (NABM)	0.6994310	0.8056306	0.9710300	0.8690500
	BMA (NBMA)	0.6194493	0.7257469	0.9716100	0.8731000
	Max (NMax)	0.2628194	0.2725935	0.9604600	0.7017200
	XGraSM-Avg (XNAvg)	**0.9649710**	**0.9963379**	**0.9816500**	**0.9204000**
	XGraSM-ABM (XNABM)	0.8500164	0.9140909	0.9747400	0.8935100
	XGraSM-BMA (XNBMA)	0.7977606	0.8885191	0.9722000	0.8758500
	XGraSM-Max (XNMax)	0.3166060	0.4174917	0.9613600	0.7065700
L	Avg (LAvg)	0.8635273	0.9838635	0.9825000	0.9181300
	ABM (LABM)	0.5666561	0.7222728	0.9716000	0.8665000
	BMA (LBMA)	0.4853167	0.6194642	0.9693300	0.8667700
	Max (LMax)	0.2174561	0.2274708	0.9606800	0.7028400
	XGraSM-Avg (XLAvg)	**0.9528206**	**0.9959297**	**0.9823900**	**0.9195700**
	XGraSM-ABM (XLABM)	0.7982155	0.8935707	0.9738000	0.8850100
	XGraSM-BMA (XLBMA)	0.7292282	0.8566720	0.9715700	0.8729400
	XGraSM-Max (XLMax)	0.3053099	0.3774761	0.9611800	0.7048300
S	Avg (SAvg)	**0.8845580**	**0.9846493**	**0.9787500**	**0.9120200**
	ABM (SABM)	0.6036674	0.7332584	0.9670700	0.8505500
	BMA (SBMA)	0.5220448	0.6330507	0.9693700	0.8665600
	Max (SMax)	0.2278649	0.2332203	0.9606000	0.7031900
Li	Avg (LiAvg)	**0.9370573**	**0.9922869**	**0.9829300**	**0.9192500**
	ABM (LiABM)	0.7209326	0.8204703	0.9685900	0.8598000
	BMA (LiBMA)	0.6436113	0.7460765	0.9698300	0.8640500
	Max (LiMax)	0.2710713	0.2850380	0.9607700	0.7032300
A	SimGIC (AGIC)	0.9173889	**0.9689432**	**0.9771400**	**0.9024400**
	SimDIC (ADIC)	0.8486233	0.9514534	0.9748200	0.8893700
	SimUIC (AUIC)	**0.9654985**	0.9654985	0.9752600	0.8937500
Z	Avg (ZAvg)	0.8628325	**0.9827186**	**0.9840200**	**0.9252700**
	ABM (ZABM)	0.5564467	0.7571073	0.9726600	0.8718700
	BMA (ZBMA)	0.4756021	0.6578980	0.9762600	0.8847200
	Max (ZMax)	0.2142027	0.2341097	0.9605400	0.7016000
	SimGIC (ZGIC)	**0.9177525**	0.9680424	0.9755300	0.8946900
	SimDIC (ZDIC)	0.8468629	0.9494608	0.9743600	0.8889200
	SimUIC (ZUIC)	0.9041007	0.9642283	0.9777800	0.9071400
W	Avg (WAvg)	0.8261012	**0.9931717**	**0.9872000**	**0.9479000**
	ABM (WABM)	0.4524186	0.8706998	0.9710900	0.8786900
	BMA (WBMA)	0.3719390	0.8287918	0.9767100	0.8861300
	Max (WMax)	0.1190595	0.2833496	0.9606800	0.7068500
	SimGIC (WGIC)	**0.8936149**	0.9659196	0.9747400	0.8909700
	SimDIC (WDIC)	0.7811533	0.9451399	0.9741200	0.8908800
	SimUIC (WUIC)	0.8678077	0.9615032	0.9733400	0.8892300
U	Avg (UAvg)	**0.9616545**	**0.9954449**	**0.9819800**	**0.9229100**
	ABM (UABM)	0.8335202	0.9513584	0.9740500	0.8819600
	BMA (UBMA)	0.7798275	0.9377088	0.9706300	0.8707200
	Max (UMax)	0.2943297	0.3982111	0.9607000	0.7050400
	SimGIC (UGIC)	0.9178478	0.9691239	0.9767900	0.9014500
	SimDIC (UDIC)	0.8758490	0.9595382	0.9751300	0.8882600
	SimUIC (UUIC)	0.9104333	0.9673649	0.9733100	0.8914700
SimUI	SimUI	0.8483416	0.9582268	0.9731600	0.8890300

Comparing performance of 57 different functional similarity measures in terms of AUC values for CC and BP ontologies, RI and NI values for the BP ontology using Protein-Protein Interaction (PPI) and Co-expression Network (CN) or Gene Expression (microarray) data. The double middle bold line separates annotation-based approaches above from the topology-based approaches below.

It was unexpected to find that the Wang et al. approach performs poorly in terms of AUC values when using the BMA and ABM measures for BP, whereas these measures have shown good performance when used in EC, Pfam domain and sequence similarity data and the authors of this approach initially suggested using the ABM measure. Other approaches show good performance when used with their initial measures even though the Avg measure achieves the best performance. On the other hand, the Max approach performs poorly compared to other approaches, independently of the network (PPI or co-expression) and performance measure. This may be due to the fact that the Max approach tends to over-estimate functional similarity scores between proteins, for example by assigning the similarity score of 1 to two proteins sharing at least one GO terms independently of the number of unrelated terms between these proteins.

Table 4 lists functional similarity measures achieving overall ‘best’ performance for different ontologies (MF, CC and BP) given a biological data type. These results indicate that for the CC ontology, the topology-based approaches, namely SimGIC based on Zhang et al. (ZGIC), Wang et al. (WGIC) and GO-universal (UGIC) measures, provide overall best performance in terms of EC, Pfam and sequence similarity, respectively. For MF and BP ontologies, annotation-based approaches, either XGraSM-Resnik BMA (XRBMA) or SimGIC (AGIC), achieve best overall best performance. This suggests that measures achieving overall best performance for EC, Pfam and Sequence Similarity data are those incorporating all informative common ancestors in their scoring systems. However, this is not the case in the context of PPI and co-expression networks where Average based on Resnik (RAvg) and Wang et al. (WAvg) measures achieve the overall best performance. If the Wang et al. approach incorporates ancestor features when modeling term semantic similarity, Resnik is based on the most informative common ancestor. To provide users with the most appropriate functional similarity measure related to the term information content or term semantic similarity approach they have chosen to use, a summary of the best performing measures for different approaches and different biological data or applications is provided in Table 5.

**Table 4:** Summary of overall ‘best’ performing measures for different biological data.

	Biological data type				
Ontology	EC	Pfam	Seq. Sim.	PPI	CN
MF	XRBMA	AGIC	AGIC		
CC	ZGIC	WGIC	UGIC	Ravg	
BP	XRBMA	XRBMA	AGIC	Ravg	Wavg

List of overall ‘best’ performing functional similarity measures for MF, CC and BP ontologies given biological data. Refer to Table 2 and 3 for the description of these different measures.

**Table 5:** Summary of the best performing measures for different applications.

Model	Approach	EC	Pfam	Seq. Sim.	PPI	CN
IC-based direct term	Annotation-based (A)	SimDIC	SimGIC	SimGIC	SimGIC	SimGIC
	GO-universal (U)	SimDIC	SimDIC	SimGIC	SimGIC	SimGIC
	Wang et al. (W)	SimGIC	SimGIC	SimGIC	SimGIC	SimGIC
	Zhang et al. (Z)	SimGIC	SimGIC	SimGIC	SimGIC	SimUIC
Pair-wise term or IC-based non direct term	Resnik (R)	BMA	Max	Max	Avg	BMA
	XGraSM-Resnik (XR)	BMA	ABM	ABM	Avg	Avg
	Nunivers (N)	BMA	ABM	ABM	Avg	Avg
	XGraSM-Nunivers (XN)	BMA	ABM	ABM	Avg	Avg
	Lin (L)	BMA	ABM	ABM	Avg	Avg
	XGraSM-Lin (XL)	BMA	ABM	ABM	Avg	Avg
	Li et al. (Li)	BMA	ABM	ABM	Avg	Avg
	Relevance (S)	BMA	ABM	ABM	Avg	Avg
	GO-Universal (U)	BMA	BMA	ABM	Avg	Avg
	Wang et al. (W)	BMA	ABM	ABM	Avg	Avg
	Zhang et al. (Z)	BMA	ABM	ABM	Avg	Avg

List of the best performing functional similarity measures, term specificity and semantic similarity approaches for different biological data, including Enzyme Commission (EC), Pfam domain, Sequence Similarity (Seq. Sim.), Protein-Protein Interaction (PPI) and Co-expression Network (CN) or Gene Expression (microarray) data.

Finally, note that the good performance of the annotation-based family is related to the corpus under consideration because of its dependence on the frequencies of GO term occurrences in the corpus. These annotations may be unbalanced in their distribution across the DAG. This constitutes a serious drawback to these approaches, specifically for organisms with sparse GO annotations and may negatively affect their performances [9]. The use of the whole set of annotations as done in this study may solve this problem but only at the cost of an increase in the running time and the complexity of these annotation-based approaches. This is expected to worsen as the number of protein annotations increases daily, which would potentially hamper the performance of these approaches in their running time, since processing the annotation file would take a lot of time before being able to compute the IC values. This implies that it is may be better to make use of topology-based approaches if one has to choose between the two families.

## Conclusion

Several IC-based GO functional similarity measures have been proposed over recent years and have enabled comparison of proteins at the functional level on the basis of their GO annotations. These measures are being used in different biological and biomedical applications and have largely contributed to the efficient exploitation of the biological knowledge embedded in the GO structure. While annotation-based functional similarity measures have been intensively studied and topology-based measures very often deployed to specific applications, none of the previous studies has attempted to quantitatively perform all-against-all semantic similarity measure comparisons. As a result, there were still gaps in our knowledge on the performance of these measures when applied to different biological data or applications, making the choice of the most ‘appropriate’ measure difficult, especially for someone who just needs a quick GO semantic similarity measure for their biological question. Thus, a comparative study was necessary in order to provide a global assessment of these different semantic similarity measures.

Here, we have carried out a quantitative performance evaluation of several different semantic similarity measures between GO terms for different term IC families or semantic similarity approaches and different biological data. Results indicate that a measure used for a given biological data type was not always the most appropriate even for the ‘well’ studied family measures, namely annotation-based measures. In fact, though the SimGIC or the BMA or ABM measure was confirmed to be the best measure, in general, when using EC, Pfam domain and sequence similarity data, this measure was not the best for applications related to PPI and co-expression data (e.g., assessing protein-protein interaction or clustering co-expressed proteins), where the Avg measure showed overall best performance. This is also the case for the topology-based approaches where, in general, the initial measure suggested for use does not provide the overall best performance. This study bridges the gap between the large variety of GO semantic similarity measures and their performance in different biological and biomedical applications by comparing different protein functional similarity measures using different biological data. This should help researchers choose the most appropriate measure for their biological application.

## Supporting Information

File S1 **Combined file of supporting tables.** Table S1: A human protein-protein interaction dataset used to assess the classification power of different functional similarity measures using Receiver Operator Characteristic (ROC) curve analysis. Table S2: A set of human protein-protein interaction with both interacting partners annotated with respect to the GO BP ontology. Table S3: A set of human protein-protein interaction with both interacting partners annotated with respect to the GO CC ontology. Table S4: A human co-expression network used to assess the clustering power of different functional similarity measures using using Normalized Mutual Information and Rank Index scores. Table S5: A set of human co-expressed protein pairs among human proteins annotated with BP terms.
(ZIP)

## References

[1] GO-Consortium (2009) The Gene Ontology in 2010: extensions and refinements. Nucleic Acids Research 38:D331–D335. 19920128 10.1093/nar/gkp1018 PMC2808930

[2] Mazandu GK, Mulder NJ (2013) DaGO-Fun: Tool for Gene Ontology-based functional analysis using term information content measures. BMC Bioinformatics 14:284. 24067102 10.1186/1471-2105-14-284 PMC3849277

[3] UniProt-Consortium (2010) The Universal Protein Resource (UniProt) in 2010. Nucleic Acids Research 38:D142–D148. 19843607 10.1093/nar/gkp846 PMC2808944

[4] Flicek P, Aken BL, Ballester B, Beal K, Bragin E, et al. (2010) Ensembl's 10th year. Nucleic Acids Research 38(Database issue): D557–D562. 10.1093/nar/gkp972 PMC2808936 19906699

[5] Sayers EW, Barrett T, Benson DA, Bryant SH, Canese K, et al. (2009) Database resources of the National Center for Biotechnology Information. Nucleic Acids Research 37(Database issue): D5–D15. 10.1093/nar/gkn741 PMC2686545 18940862

[6] Benson DA, Karsch-Mizrachi I, Lipman DJ, Ostell J, Sayers EW (2009) Genbank. Nucleic Acids Research 37(Database issue): D26–D31. 10.1093/nar/gkn723 PMC2686462 18940867

[7] Mazandu GK, Mulder NJ (2012) Using the underlying biological organization of the *Mycobacterium tuberculosis* functional network for protein function prediction. Infection, Genetics and Evolution 12(5):922–932. 10.1016/j.meegid.2011.10.027 22085822

[8] Mazandu GK, Mulder NJ (2013) Information content-based Gene Ontology semantic similarity approaches: Toward a unified framework theory. BioMed Research International 2013: Ariticle ID 292063, 11 pages. 10.1155/2013/292063 PMC3775452 24078912

[9] Mazandu GK, Mulder NJ (2012) A topology-based metric for measuring term similarity in the Gene Ontology. Adv Bioinformatics 2012: Ariticle ID 975783, 17 pages. 10.1155/2012/975783 PMC3361142 22666244

[10] Guzzi PH, Mina M, Guerra C, Cannataro M (2011) Semantic similarity analysis of protein data: assessment with biological features and issues. Brief Bioinform: 1–17. 10.1093/bib/bbr066 22138322

[11] Pesquita C, Faria D, Falcão AO, Lord P, Couto FM (2009) Semantic similarity in biomedical ontologies. PLoS Comput Biol 5(7):e1000443. 19649320 10.1371/journal.pcbi.1000443 PMC2712090

[12] Mistry M, Pavlidis P (2008) Gene Ontology term overlap as a measure of gene functional similarity. BMC Bioinformatics 9:327. 18680592 10.1186/1471-2105-9-327 PMC2518162

[13] Resnik P (1999) Semantic similarity in a taxonomy: An information-based measure and its application to problems of ambiguity in natural language. Journal of Artificial Intelligence Research 11:95–130.

[14] Lin D (1998) An information-theoretic definition of similarity. In: Proceedings of the Fifteenth International Conference on Machine Learning. pp.296–304.

[15] Jiang JJ, Conrath DW (1997) Semantic similarity based on corpus statistics and lexical taxonomy. In: Proceedings of the 10th International Conference on Research in Computational Linguistics. pp.19–33.

[16] Lord PW, Stevens PW, Brass A, Goble CA (2003) Investigating semantic similarity measures across the Gene Ontology: the relationship between sequence and annotation. Bioinformatics 19(10):1275–1283. 12835272 10.1093/bioinformatics/btg153

[17] Couto F, Silva M, Coutinho P (2007) Measuring semantic similarity between Gene Ontology terms. Data Knowledge Eng 61(1):137–152.

[18] Schlicker A, Domingues FS, Rahnenfuhrer J, Lengauer T (2006) A new measure for functional similarity of gene products based on Gene Ontology. BMC Bioinformatics 7:302. 16776819 10.1186/1471-2105-7-302 PMC1559652

[19] Li B, Wang JZ, Feltus FA, Zhou J, Luo F (2010) Effectively integrating information content and structural relationship to improve the GO-based similarity measure between proteins. ArXiv e-prints: 1001.0958.

[20] Yang H, Nepusz T, Paccanaro A (2012) Improving GO semantic similarity measures by exploring the ontology beneath the terms and modelling uncertainty. Bioinformatics 28(10):1383–1387. 22522134 10.1093/bioinformatics/bts129

[21] Sevilla JL, Segura V, Podhorski A, Guruceaga E, Mato JM, et al (2005) Correlation between gene expression and GO semantic similarity. IEEE/ACM Transactions on Computational Biology and Bioinformatics (TCBB) archive 2(4):330–338. 10.1109/TCBB.2005.50 17044170

[22] Pesquita C, Faria D, Bastos H, Ferreira AEN, Falcão AO, et al**.** (2008) Metrics for GO based protein semantic similarity: a systematic evaluation. BMC Bioinformatics 9(Suppl 5) S4. 10.1186/1471-2105-9-S5-S4 PMC2367622 18460186

[23] Gentleman R (2005) Visualizing and Distances Using GO, http://bioconductor.org/packages/2.6/bioc/vignettes/GOstats/inst/doc/GOvis.pdf.

[24] Wang JZ, Du Z, Payattakool R, Yu PS, Chen CF (2007) A new method to measure the semantic similarity of GO terms. Bioinformatics 23(10):1274–1281. 17344234 10.1093/bioinformatics/btm087

[25] Zhang P, Jinghui Z, Huitao S, Russo JJ, Osborne B, et al (2006) Gene functional similarity search tool (GFSST). BMC Bioinformatics 7:135. 16536867 10.1186/1471-2105-7-135 PMC1421445

[26] Seco N, Veale T, Hayes J (2004) An intrinsic information content metric for semantic similarity in wordnet. In: ECAI-04. pp. 1089–1090.

[27] Wu X, Pang E, Lin K, Pei Z (2013) Improving the Measurement of Semantic Similarity between Gene Ontology Terms and Gene Products: Insights from an Edge- and IC-Based Hybrid Method. PLoS ONE 8(5):e66745. 23741529 10.1371/journal.pone.0066745 PMC3669204

[28] Xu Y, Guo M, Shi W, Liu X, Wang C (2013) A novel insight into Gene Ontology semantic similarity. Genomics 101:368–375. 23628645 10.1016/j.ygeno.2013.04.010

[29] Alvarez MA, Qi X, Yan C (2011) A shortest-path graph kernel for estimating gene product semantic similarity. J Biomed Semant 2:3. 10.1186/2041-1480-2-3 PMC3161911 21801410

[30] Pesquita C, Faria D, Pessoa D, Couto FM (2009) CESSM: Collaborative Evaluation of Semantic Similarity Measures. JB2009: Challenges in Bioinformatics 157.

[31] Bossi A, Lehner B (2009) Tissue specificity and the human protein interaction network. Molecular Systems Biology 5:260. 19357639 10.1038/msb.2009.17 PMC2683721

[32] Mazandu GK, Opap K, Mulder NJ (2011) Contribution of microarray data to the advancement of knowledge on the *Mycobacterium tuberculosis* interactome: Use of the random partial least squares approach. Infection, Genetics and Evolution 11(4):725–733. 10.1016/j.meegid.2011.04.012 21514402

[33] Blondel VD, Guillaume JL, Lambiotte R, Lefebvreet E (2008) Fast unfolding of communities in large networks. J Stat Mech 10008:1–12.

[34] Steinhaeuser K, Chawla NV (2010) Identifying and evaluating community structure in complex networks. Pattern Recognition Letters 31:413–421.

